# Analysis of World Economic Variables Using Multidimensional Scaling

**DOI:** 10.1371/journal.pone.0121277

**Published:** 2015-03-26

**Authors:** J.A. Tenreiro Machado, Maria Eugénia Mata

**Affiliations:** 1 Institute of Engineering, Polytechnic of Porto, Dept. of Electrical Engineering, Porto, Portugal; 2 Nova SBE, Universidade Nova de Lisboa, Faculdade de Economia, Campus de Campolide, Lisbon, Portugal; Arizona State University, UNITED STATES

## Abstract

Waves of globalization reflect the historical technical progress and modern economic growth. The dynamics of this process are here approached using the multidimensional scaling (MDS) methodology to analyze the evolution of GDP per capita, international trade openness, life expectancy, and education tertiary enrollment in 14 countries. MDS provides the appropriate theoretical concepts and the exact mathematical tools to describe the joint evolution of these indicators of economic growth, globalization, welfare and human development of the world economy from 1977 up to 2012. The polarization dance of countries enlightens the convergence paths, potential warfare and present-day rivalries in the global geopolitical scene.

## Introduction

Historical facts may suggest that democracy is endogenous to political regimes. The victory of the Allied Nations in 1945 led to the creation of the United Nations to preserve peace and respect for human rights, as conditions for the economic growth of nations. The political ideals of Churchill and Roosevelt included not only sustainable peace but also sustainable economic growth and prosperity for all.

The first key ingredient of economic growth was reconstruction and recovery. The Marshall Plan expressed the American help to European partners to promote the adoption of technological innovation and imitation that would multiply positive effects to support economic growth.

The global post-war perspective was based on optimistic views on growth and democracy. A considerable analytical effort to analyze these aspects was framed in the context of job opportunities and modernization for reaping the respective spillovers of modernization. Convergence was the dominant view, and economic growth was forecasted, based on decreasing marginal capital returns and large availability of economic factors and resources, such as population growth, large labor force, savings, capital, and transfers of technology [1]. Within the New International Economic Order (NIEO) hopes on economic growth also led to the United Nations’ declaration of January 1961 that the 1960s would be “a decade for growth and development”.

The market virtues of capitalism framed the ideological platform of the Western World on the effects of foreign trade openness and global partnerships, to include free movements of capital and foreign direct investment among all countries [2]. In 1964 the United Nations Conference on Trade and Development (UNCTAD) again declared the 1970s as a new “decade for growth and development” [3]. Multiplier effects were crucial to understand the evolution of prosperity and welfare during the Golden Age of 1945-1974. The less developed an economy was at the outset of economic development, the greater speed it would show in pursuing growth, catching-up with more developed nations, to cancel any divergence from the most developed countries [4].

The extant literature in economic history, including iconic books on poverty and prosperity were (and still are) amazing sources of optimistic good-will [5], and reports the variables that may command and launch the global growth and prosperity [6], both in the USA and in Africa [7–10]. Supposing that consumption preferences command demand and production, countries are very homogeneous in pressing politicians and economists to accomplish their social mission as economic growth supporters, in order to spread prosperity and welfare. International division of labor was meant to lead international solidarity and schooling in modern economies, using sophisticated technological systems, which endogenously should produce the human capital that was required for longevity [11] under social welfare [12].

The purpose of this paper is to address the analysis of the dynamics of countries as complex systems. It seeks to assess similarities and dissimilarities among national economies in the historical process. Some of them pioneered industrialization, and some others were latecomers in achieving economic growth, mass consumption and economic maturation. How successful was convergence? Using Multidimensional Scaling (MDS) in order to visually check the proximity and similarities among countries, this paper covers the last 36 years, using a database of 14 countries. The new millennium brought financial crises that remember the Great Depression of 1929-33 [13, 14].

One may recall 2002 in Argentina, 2004 in Japan, 2007 in the USA, 2009 in Ireland, 2010-13 in Greece, 2011-14 in Portugal, and 2012-13 in Spain and Italy [15]. The world fears global imbalances for economic growth, welfare convergence [16, 17] and democracy, especially after 2040 [18]. What can MDS methodology tell us on that [19, 20]?

In this line of thought the paper is organized as follows. Section 2 reviews the literature, while Section 3 presents the data details and the methodology in the study. Section 4 discusses the MDS results, namely the interpretation of maps for the distinct experiments. Finally, Section 5 outlines the main conclusions.

## Approaches on theoretical and methodological issues

The MDS methodology allows an effective description of the polarization dance of the analyzed countries to check convergence [21]. According to Neoclassical economics, there is a negative relationship between the growth rate and the initial level of income. For the *convergence hypothesis*, the speed of convergence for countries having low amounts of capital is predicted to be faster, in the economic growth process [22]. Literature even reports convergence for OECD countries’ productivity in the 1950-85 period [23].

According to endogenous growth theories [24], no evidence on these conclusions could be quoted for a sample of 100 countries if standards of life were measured according to purchasing power parity (PPP) values [25]. Moreover, besides the initial level of revenue, other variables proved to be important to define growth and prosperity, using a similar sample of countries [26]. It was even possible to demonstrate that a small number of variables were significant in explaining growth using an econometric cross-sectional analysis [27]. Variables including regional, political, sociological, and global shocks were also used in long regressions to check convergence [28, 29].

A 2% rate of convergence was discovered for databases, opening hopeful perspectives for a convergence process [30] in 35 years. Such a period would be enough to cancel half of the distance of a country to its steady-state [30]. If such a process would go on, 70 years would cancel the whole distance to a steady state condition [31], particularly if capital loans could flow easily [32]. It was discovered that in 2000 there were 500 million fewer poor than in 1970 [33], particularly because of growth in China and India [29]. Everywhere longevity proved large life expectancy gains [34], especially in OECD countries [12].

Can the current European financial crisis prove that convergence has been an over-optimistic hypothesis? For the new millennium? For the whole period?

## Data and methodology

The database for 14 countries includes GDP per capita [33], openness (given by the percentage of exports in GDP), life expectancy, and education tertiary enrollment for a 36 years time-span. Data was collected from the *World Bank national development indicators* which cover exactly the period coming from 1977 to 2012 (source http://data.worldbank.org/data-catalog/world-development-indicators from 1970 onward for education).

The economic variables under analysis are:

- GDP per capita, comes from NY.GNP.PCAP.KD. It is the Gross National Income (GNI) per capita (constant 2005 US$). GNI per capita is gross national income divided by midyear population. GNI (formerly GNP) is the sum of value added by all resident producers plus any product taxes (less subsidies) not included in the valuation of output plus net receipts of primary income (compensation of employees and property income) from abroad. Data are in constant 2005 U.S. dollars.
- Annual exports of goods and services (% of GDP) comes from NE.EXP.GNFS.ZS. Exports of goods and services represent the value of all goods and other market services provided to the rest of the world. They include the value of merchandise, freight, insurance, transport, travel, royalties, license fees, and other services, such as communication, construction, financial information, business, personal, and government services. They exclude compensation of employees and investment income (formerly called factor services) and transfer payments. It is a weighted average. Data are expressed in percentage of GDP.
- Life expectancy comes from SP.DYN.LE00.IN. Life expectancy at birth indicates the number of years a newborn infant would live if prevailing patterns of mortality at the time of its birth were to stay the same throughout its life.
- School enrollment, tertiary (% gross) comes from SE.TER.ENRR. Gross enrollment ratio is the ratio of total enrollment, regardless of age, to the population of the age group that officially corresponds to the level of education shown. Tertiary education, whether or not to an advanced research qualification, normally requires, as a minimum condition of admission, the successful completion of education at the secondary level. Data are expressed in percentage. The time series for tertiary enrollment is the least complete, but all education time series present missing observations. Barro & Lee [35] database for 1950-2010 is an excellent source for education, but the information is only given for every five years. The UNESCO education database has yearly information, but only covers 1970-1997, with missing points.

For treating this large volume of data we adopt MDS which is a computational, statistical, and visualization technique that produces a representation of “similarity” between objects [21, 36, 37].

The MDS representation of *n* objects requires the definition of a measure *δ*_*ij*_ for distance between items *i* and *j*, *i*, *j* = 1, ⋯, *n*, followed calculation of a *n*×*n* symmetrical matrix **Δ** measuring the distance between all pairs of objects:

$$\begin{array}{c} \Delta =\left[\begin{array}{ccc} \delta_{11} & \cdots & \delta_{1n}\\ \vdots & \ddots & \vdots \\ \delta_{nn} & \cdots & \delta_{nn} \end{array}\right] \end{array}$$ (1)

Given a distance *δ*_*ij*_, MDS tries to obtain the position vectors *x*_*i*_ and *x*_*j*_ such that the vector norm *d*_*ij*_ = ∣*x*_*i*_−*x*_*j*_∣ is close to *δ*_*ij*_. By other words, MDS represents an optimization problem, where vectors {*x*_1_, …, *x*_*n*_} are found by minimizing some kind of cost function, often called “stress” *S*, such as:

$$\begin{array}{c} \min_{x_{1},\cdots ,x_{n}}\sum_{i<j}{\left(d_{ij}-\delta_{ij}\right)}^{2} \end{array}$$ (2)

If the distance between two objects is zero/infinite, then corresponding MDS points are superimposed/very far apart. Inversely, if two points are located closely/far apart in the MDS plot, then there is a small/large distance between the corresponding data vectors [38]. Instead of distances we can use some correlation measure and then the interpretation follows the inverse path, namely, two objects with high/low correlation produce two points close/distant in the MDS map.

MDS executes a numerical optimization procedure to estimate the coordinates of the points in an *m*-dimensional map, based on matrix **Δ** storing the distances (to be defined in some mathematical sense) between all pairs of objects [39]. Often it is considered *m* = 2 or *m* = 3, simply because it leads to a direct graphical visualization of the MDS map.

The symmetric matrix **Δ** = [*δ*_*ij*_] main diagonal is composed of zeros, while the rest of the matrix elements must obey the restriction *δ*_*ij*_ ≥ 0, *i*, *j* = 1, ⋯, *n*. Since MDS works with relative measurements, the resulting maps are not sensitive to translations or rotations and the axes have only the meaning and units (if any) of the measuring index. MDS rearranges objects in order to produce a map that best approximates the observed similarities. A measure for evaluating the accuracy of the MDS solution is the raw stress *S*. The smaller the stress value *S*, the better is the fit. Plotting the stress versus the number of dimensions *m* of the MDS map produces a monotonic decreasing chart, sometimes called “scree plot”. The user chooses the “best” dimension as a compromise between stress reduction and number of dimensions for the map representation. The quality of MDS plot can be also accessed by means of the Shepard plots that represents input distances *δ*_*ij*_ against output distances (MDS produced) *d*_*ij*_ for every pair of items and a given dimension *m* of the MDS representation.

MDS software is often referred to in the literature as a statistical tool, but its main characteristic is that it provides a means of *visualizing* items without requiring additional constraints, or by defining *a priori* restrictions. The MDS map reflects the properties of the similarity measures and distinct indices produce different maps. Therefore, MDS charts lead to a direct visualization of results that can also be obtained by the combination of the same analytical techniques with the expense of a more laborious comparison.

Several programs implement MDS directly and we can mention Matlab [40, 41], R [42, 43] and GGobi [44, 45].

In our case the objects are points in the MDS map and represent vectors of data describing economic variables. Here the objects are country economies characterized by means of a given set of variables evaluated during a given time period.

The classical MDS does not explicitly include a description of time/space evolution. Capturing time/space dynamics may be embedded indirectly into the correlation measure as long the comparison index between variables has some built-in feature. For example, a histogram-based correlation discharges time dependent phenomena. For highlighting time dynamics explicitly, the division of the total time period of analysis into several time periods of width *h* to be treated by MDS as independent objects was proposed. Consequently, for a total time period *T*, this method produces *p* = *T*/*h* samples and the number of MDS points increases proportionally. In this line of thought, the time samples to be adopted are a compromise between capturing fast dynamics (possible only with small values of *h*), and producing a limited number of MDS points (which requires large values of *h*). Available data allows us to consider time series along a period *T* = 36 years, namely from 1977 up to 2012. In the sequel two cases are considered, namely a comparison based on the whole period of time, that is *h* = 36 (i.e., *p* = 1) and *h* = 12 (i.e., *p* = 3). Furthermore, a set of *n*_*c*_ = 14 countries is tackled, namely {ARG, AUS, BRA, CAN, CHN, FRA, DEU, IND, ITA, JPN, MEX, RUS, GBR, USA} ≡ {Argentina, Australia, Brazil, Canada, China, France, Germany, India, Italy, Japan, Mexico, Russian Federation, UK, US}. We obtain *n* = *n*_*c*_×*p* objects to be analyzed in the MDS, of *h* years length each. Therefore, the two cases consist of *p* = 3, *h* = 12, *n* = 42 and *p* = 1, *h* = 36, *n*_*c*_ = 14. In the chart, points labeled as “AUS1” or “USA3” mean Australia during the first period (1977-1988) and United States during the last (third) period (2001-2012), respectively. As mentioned above *l* = 4 economic variables, namely {GDP per capita, openness, life expectancy, education tertiary enrollment}, are adopted for characterizing each country economy, having identical weights.

**Fig 1:**
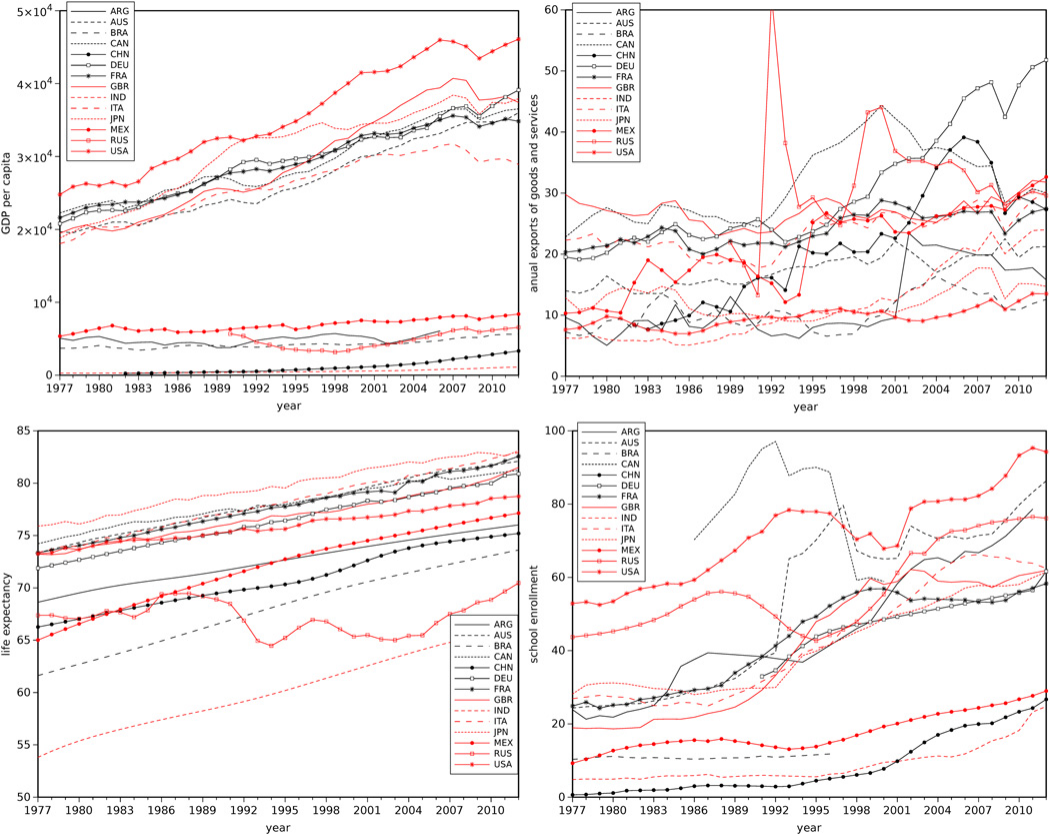
Time evolution of the 4 selected variables for the set of 14 countries during 1977-2012.

The data set of four economic variables had some handling difficulties, because in several cases values were missing (see Fig. 1). Several countries had some periods without data values that had to be estimated by means of linear interpolation between adjacent years. In particular the Russian Federation, for all variables, and Germany for the education tertiary enrollment had many missing values. More complex to tackle were missing values at the beginning or end of the 1977-2012 period, since there were no extreme supporting values for some kind of interpolation. Extensive tests with trendlines revealed that it was necessary to adopt distinct functions for each case in order to obtain a reasonable estimate (where “reasonable” means that a visual inspection and comparison with the other countries seemed adequate). Since such heuristic procedure is difficult to replicate we decided to adopt a distinct technique. Therefore, when evaluating distances between two items (to be described in the sequel) are only considered as valid points those that have numerical data.

For constructing matrix **Δ** = [*δ*_*ij*_] two alternative indices are adopted, namely the Cosine correlation and the Canberra distance, defined as:

$$\begin{array}{c} \delta_{ij}=1-\frac{\sum_{t}\sum_{k}x_{i}\left(k,t\right)x_{j}\left(k,t\right)}{\sqrt{\sum_{t}\sum_{k}x_{i}^{2}\left(k,t\right)\cdot \sum_{t}\sum_{k}x_{j}^{2}\left(k,t\right)}},\;i,j=1,\cdots ,n, \end{array}$$ (3)

$$\begin{array}{c} \delta_{ij}=\frac{1}{n_{s}}\sum_{t}\sum_{k}\frac{\left|x_{i}\left(k,t\right)-x_{j}\left(k,t\right)\right|}{\left|x_{i}\left(k,t\right)\right|+\left|x_{j}\left(k,t\right)\right|},\;i,j=1,\cdots ,n, \end{array}$$ (4)

where *x*_*i*_ and *x*_*j*_ are economic variables for the *i*-th and *j*-th objects, *t* and *k* denote two dummy indices representing time and type of economic variable, and *n* denotes the total number of objects. Equation (3) is the normalized inner product and is often called the cosine coefficient because it measures the angle between two vectors, denoting an angular metric [39, 46]. Equation (4), where *n*_*s*_ denotes the number of valid pairs of points, is inspired in the average Canberra distance over the time period. For both indices, distances are calculated only for pairs of valid points, avoiding the problem of missing values in the extremes of the data series for some countries. In general, sums are calculated for 1 ≤ *t* ≤ *h* and 1 ≤ *k* ≤ *l*, with exception of not-valid pairs of points, and the indices adjust the resulting final measure since sums are present both in numerator and denominator. By other words, both expressions yield relative values, that is, compare series without being sensitive to scale factors, the problem of lack of information is diminished.

The time-based approach seems to be appropriate for capturing a picture of the relative similarities among countries. Shorter periods would allow for more time detail, but the number of points in the plots would increase, making them more difficult to read. The 12-years approach also may help in comprising any Juglar business-cycle behavior that may influence the countries’ achievements. Furthermore, expressions (3) and (4) have an embedded implicit description of the time evolution, which would not be possible with other measures such as, for example, histograms. Therefore, cases *p* = 3 and *p* = 1 differ simply because they reflect time dynamics more or less explicitly.

## The MDS results

According to Fig. 1 the variable GDP per capita may lead to distinguish two groups of countries, as low-revenue per capita countries such as Argentina, Brazil, Mexico, Russia, India or China may be considered as much poorer than some others such as the USA, the United Kingdom and Germany. Life expectancy reveals some of these differences among the countries here analized, and the Russian 1980s illustrate the difficulties resulting from the end of communism and the move to capitalism, through the introduction of a market economic system. The Russian life expectancy was moving according to a positive trend, as in capitalist countries, but declined in a significant way in the next years, to only recover in the new millennium, although it still remains below the Western world top performances. The 1992 strange value for Russian exportation openness does not correspond to a statistical transcription error we have committed, but is certainly a heterogeneous estimation (probably a change in the country criteria for estimations). Countries’ tertiary school enrollments reflect the GDP per capita ranking, and the evolutionary path of this variable may be related with business cycle fluctuations (one should note that the 1986-98 years have been a very hopeful period for most of the countries).

Did the relative proximities among countries change throughout the historical process here analyzed? What national cases were the most similar when looking at the entire 36 years of economic growth? Can MDS express economic development, social welfare and potential warfare?

A different label with three letters and one number signals the results for each country and 12-years time period, namely {ARG, AUS, BRA, CAN, CHN, FRA, DEU, IND, ITA, JPN, MEX, RUS, GBR, USA} followed by the numbering {1,2,3} for 1977-1988, 1989-2000 and 2001-2012, respectively (*p* = 3). In the case of considering all time period of 36 years (*p* = 1) no number is attached to the three letters. Results show relative positions among countries, for each of the clusters that represent each 12-year period. Therefore, neighboring means high similarity, and distance means the opposite.

**Fig 2:**
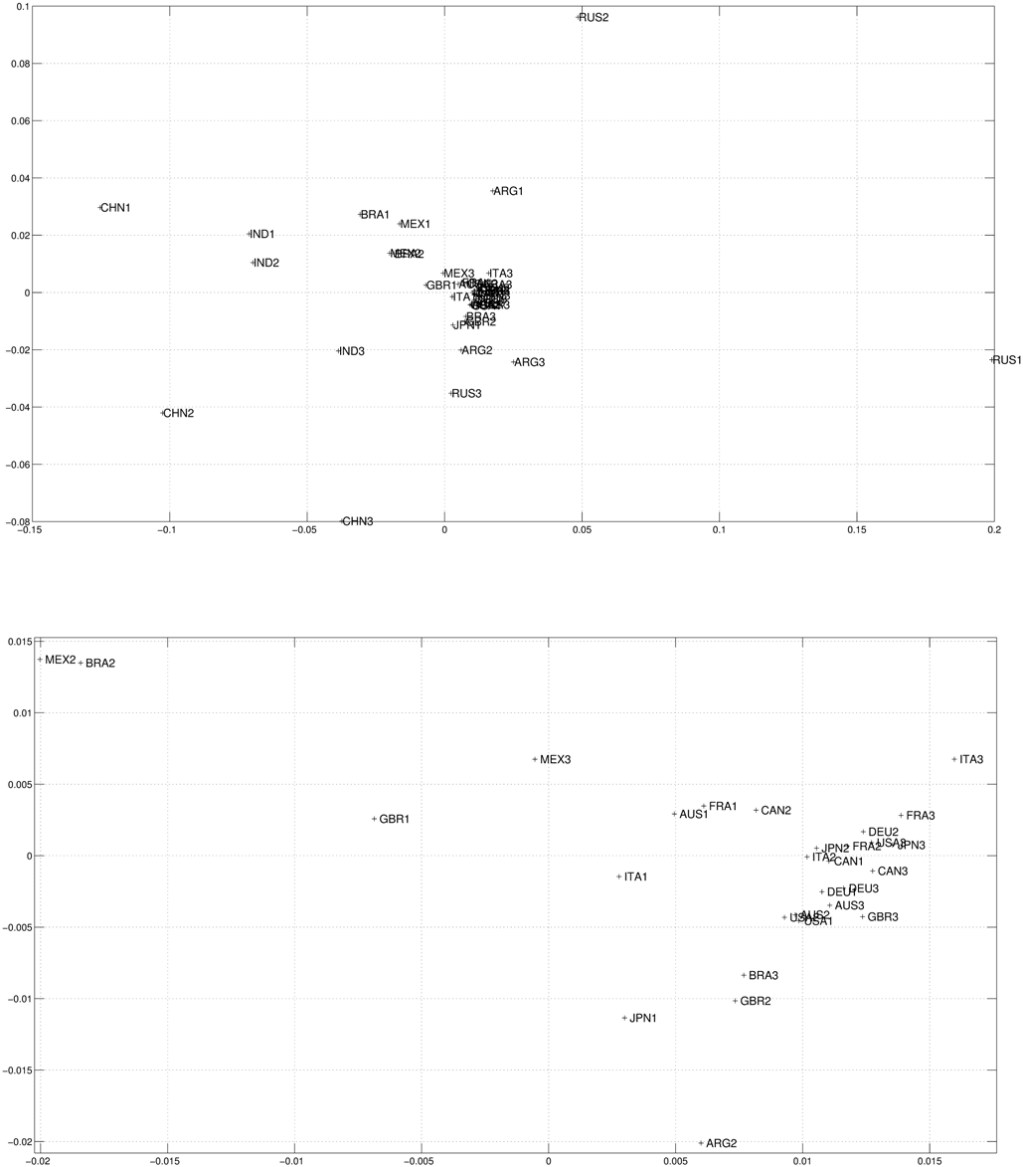
Two dimensional MDS map using Cosine-correlation (3) for the *n*_*c*_ = 14 countries and 3 periods (i.e., *m* = 2, *p* = 3), based on the 4 selected variables. Total map (top) and magnification (bottom) of the central area.

Human welfare is a multidimensional aspect of collective life, here approached for 14 large world economies, for the last 36 years, through longevity (life expectancy at birth), openness, income per capita, and education (tertiary education enrollment). Substantial gains of wellbeing are observed for all the countries analyzed. In the seventies, the Latin American Mexico, Brazil and Argentina were quite similar and formed a node in Fig. 2 top. This fact may recall the literature on clubs of convergence, but this is not the issue examined in this paper. China, India and Russia were very dissimilar. In the next 12-year periods, catching-up with European and North-American partners has been the rule. Socialism or capitalism, having different policies for education, technology, health, etc., converged. Differentiated cultural aspects related with long-rooted Asian civilizations (in India and China) did not prevent economic success and catching-up. Catching-up has occurred mainly over the two last 12-year periods, thanks to the Asian performance driven by the Chinese and the Indian economies, whose dimension may threaten other global partners. (See Fig. 2 top, which has a second chart in bottom to magnify the node of countries in the central part of the first picture that strongly converged, making an unreadable cloud of similarity partners).

**Fig 3:**
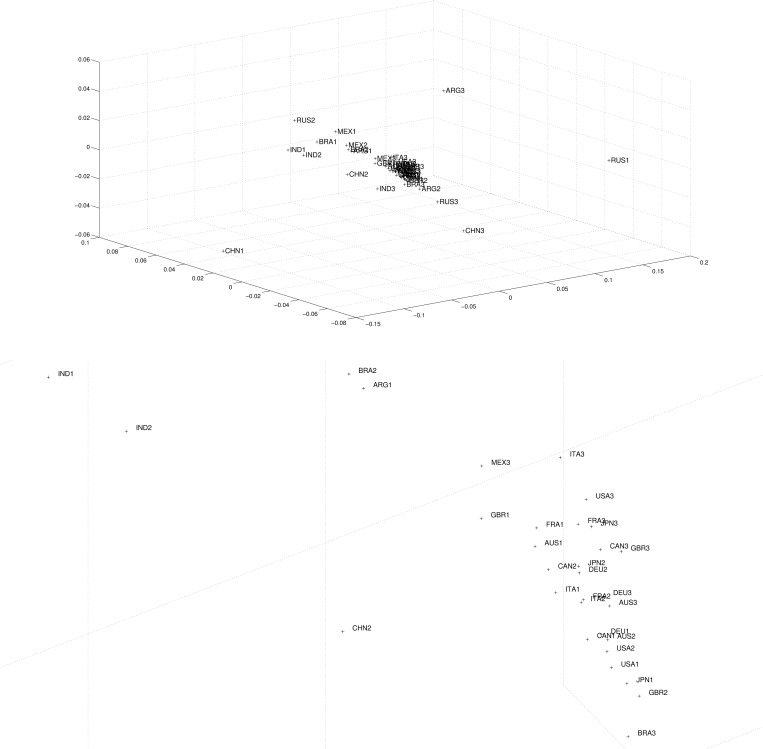
Three dimensional MDS map using Cosine-correlation (3) for the *n*_*c*_ = 14 countries and 3 periods (i.e., *m* = 3, *p* = 3), based on the 4 selected variables. Total map (top) and magnification (bottom) of the central area.

Quality of life has improved in a sustained way with large progress for the humankind in the context of globalization. The use of a three-dimension picture in Fig. 3 top confirms that catching-up has occurred mainly in the second 12-year period and in the new millennium. The amplification of the central node of countries that remained unreadable in Fig. 3 top can distinguish the most remarkable welfare-level countries of the world in Fig. 3 bottom.

Countries reveal peculiar paths. The UK was the most precocious country to take off in the 1780s [2]. France was following in the 1810s, the United States and Germany in the 1840s, Japan and Russia in the 1880s, Italy and Canada in the 1890s, Australia in the 1900s, Argentina and Brazil in the 1920s, Mexico in the 1940s, India, and China in the 1950s [2].

Convergence prevailed, and neighborhoods changed. This indicates that historical events such as Japan’s defeat in the Second World War were not detrimental enough to prevent that country from occupying a position close to its nineteenth-century industrializing partners, three decades after the end of the conflict and two decades after the end of the American military occupation of Japan, which lasted until 1953. In fact, the presence of American multinationals and the quick industrial recovery in Japan led this country to achieve a position quite close to other old partners, something that historians have labeled as “a Japanese miracle”.

**Fig 4:**
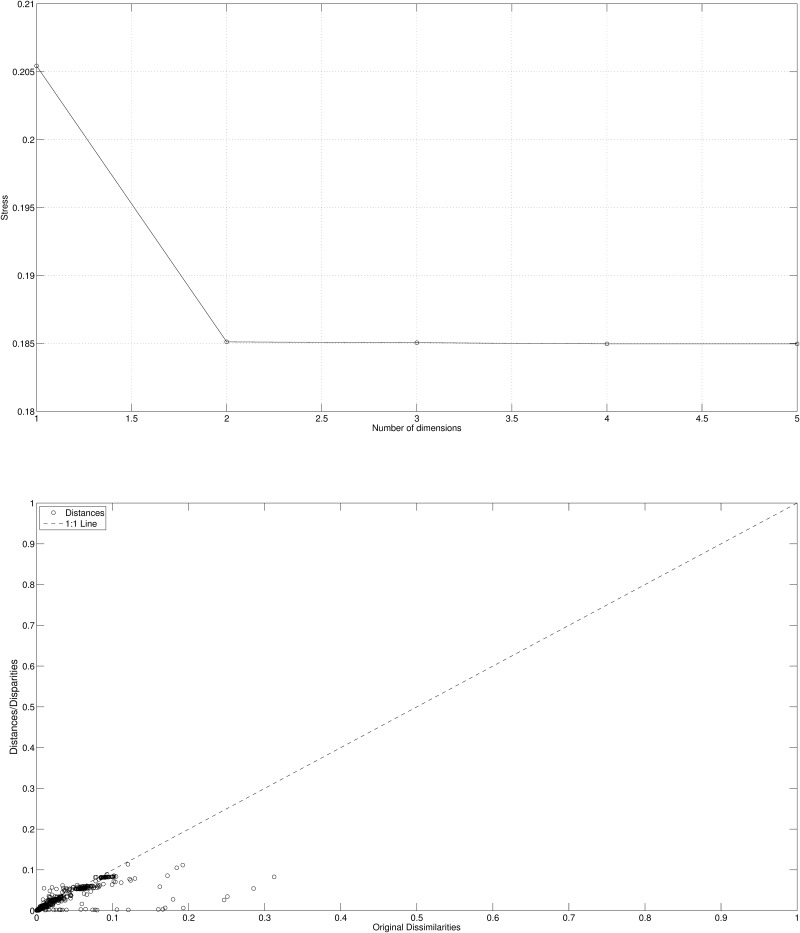
Stress plot (left) and Shepard diagram for *m* = 2 (right) using Cosine-correlation (3) for the *n*_*c*_ = 14 countries and 3 periods (i.e., *p* = 3), based on the 4 selected variables.

The quality of MDS plot is accessed often by means of the stress and the Shepard plots that are represented in Fig. 4.

The stress plot reveals that *m* = 2 establishes a good compromise between accuracy and visualization simplicity.

**Fig 5:**
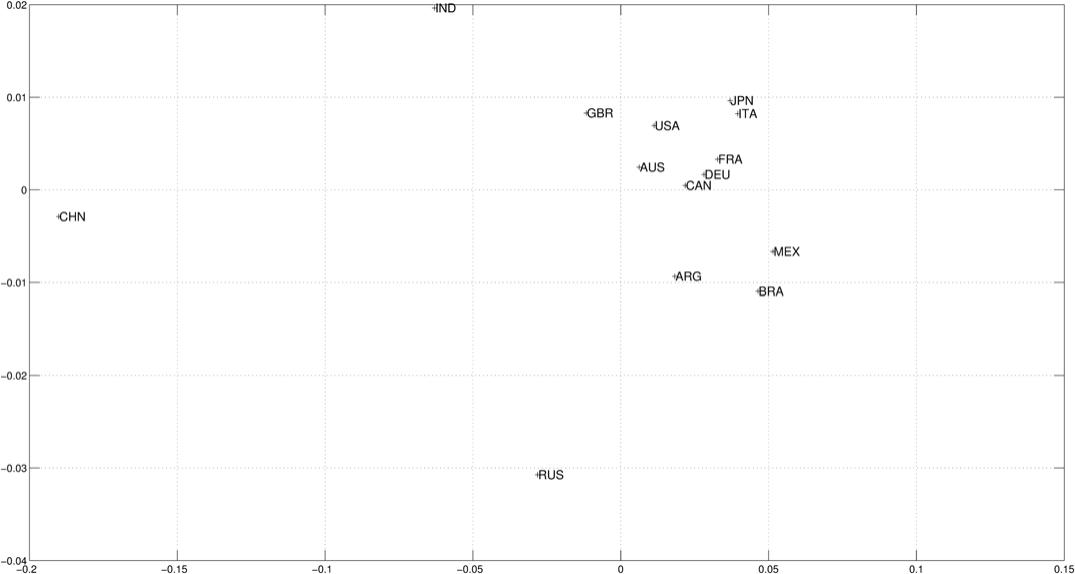
Two dimensional MDS map using Cosine-correlation (3) for the *n*_*c*_ = 14 countries and the whole period (i.e., *m* = 2, *p* = 1), based on the 4 selected variables.

For capturing the path as a whole, Fig. 5 represents the MDS map using Cosine-correlation distances among the 14 partners in the entire period.

Fig. 5 evokes the proximity among the sampled countries. The mortality reduction through the decrease of infect-contagious disease has represented the victory of hope over death, everywhere. International trade and openness in general have provided the conditions for an international specialization according to local abilities and comparative advantages, bringing an international division of labor. For education, tertiary literacy combined with higher income per capita have established a mutual influence going from higher revenues to larger enrollments in tertiary education, and from tertiary education technical abilities to higher efficiency, productions, and revenues.

In the last 36 years as a whole, a quick catching-up process occurred in large areas of Asia and Latin America. The Western World (made of European and North-American partners) has experienced economic growth at a lower pace in the new millennium. Fig. 5 shows that Latin-American latecomers Mexico, Argentina and Brazil are very close to any other country. Having late take-off experiences, they clearly converged to Canada and the European countries that experienced earlier take-off industrializations. These decades under observation have been the right time for their maturation, and they all should be classified as developed mass-consumption regions, according to historians’ 1960s expectations [2].

**Fig 6:**
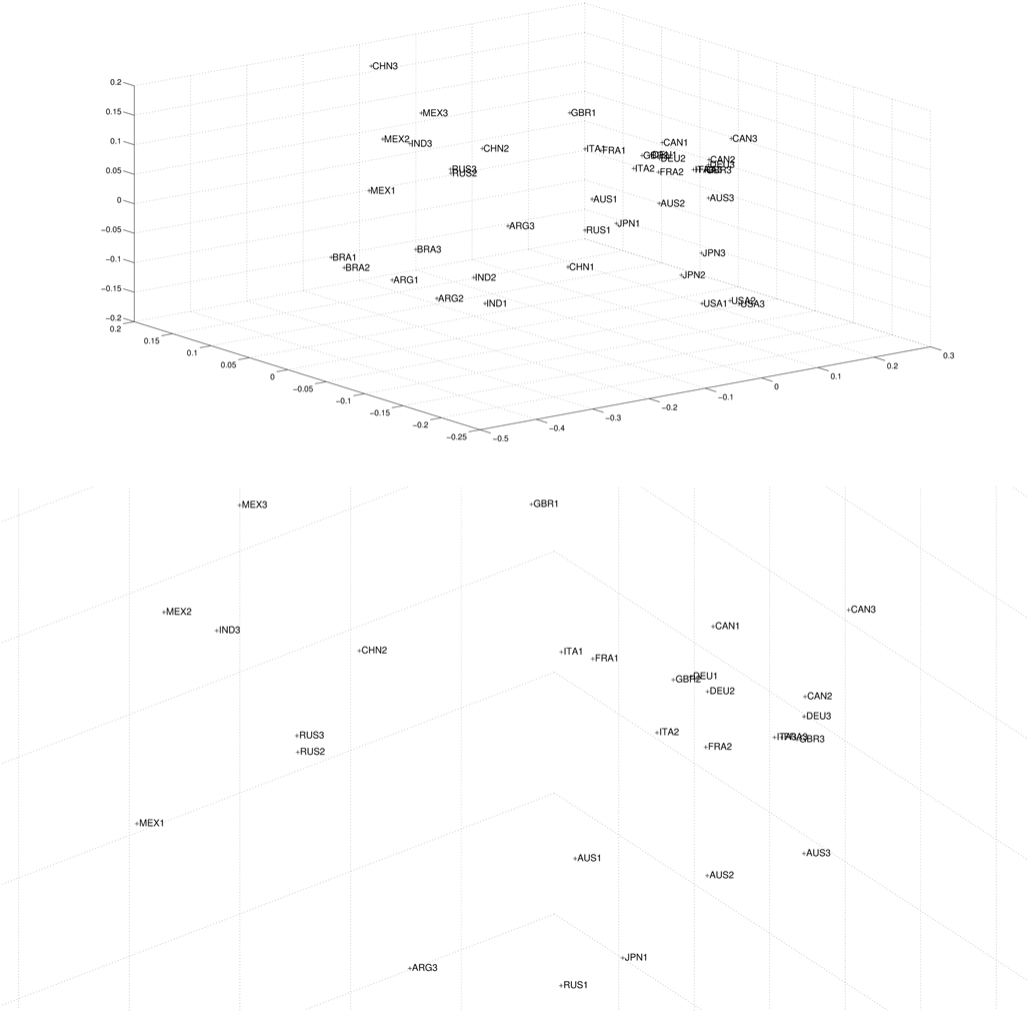
Three dimensional MDS map using Canberra distance (4) for the *n*_*c*_ = 14 countries and 3 periods (i.e., *m* = 3, *p* = 3), based on the 4 selected variables. Total map (top) and magnification (bottom) of the central area.

The exercise might finish here, but it is worth mentioning that conclusions are confirmed if Canberra distance is used. All estimations were repeated, this time using Canberra distances, to make the exercise more robust and conclusions more reliable. They are available in the next four charts. Fig. 6 top presents a tri-dimensional MDS chart for the 3 periods (i.e., *m* = 3, *p* = 3) using Canberra distance (4) for the 14 countries. A small number of countries have special positions of their own, confirming the conclusions from Fig. 2. Fig. 6 bottom amplifies three nodes of countries that were unreadable in Fig. 6 top, and confirms the top welfare positions of the North-American and European countries.

**Fig 7:**
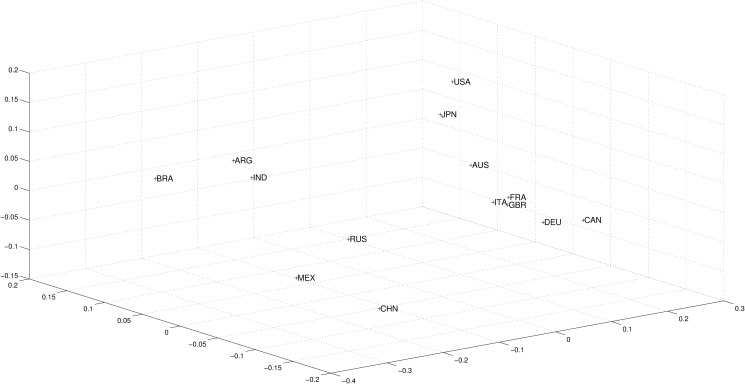
Three dimensional MDS map using Canberra distance (4) for the *n*_*c*_ = 14 countries and the whole period (i.e., *m* = 3, *p* = 1), based on the 4 selected variables.

We now return to analyzing the 36 year period as a whole. Fig. 7 presents the MDS map using the Canberra distance. Previous conclusions seem to be clearer. In the same way, one may distinguish a cloud of similarity, comprising countries that converged in spite of their different take-off dates. The Russian convergence with the Western-world economies is confirmed, as proximity to them is much closer than for Asian partners. The new different position for Russia stands out in the period of deep reforms away from communism and central planning. The conclusions stand, using both the cosine-correlation (3) and the Canberra distance (4). At the same time, the image of the 14 analyzed countries, when depicted on a plane after MDS calculations, appear similar.

The new-millennium European crisis, combined with successful Asian industrializations, and the extension of market mechanism to a global world, free of central-planning systems, has offered hopeful new opportunities for convergence, although the global market disclosure is revealing high uncertainty in the Old World, particularly because of instability in the Euro zone.

**Fig 8:**
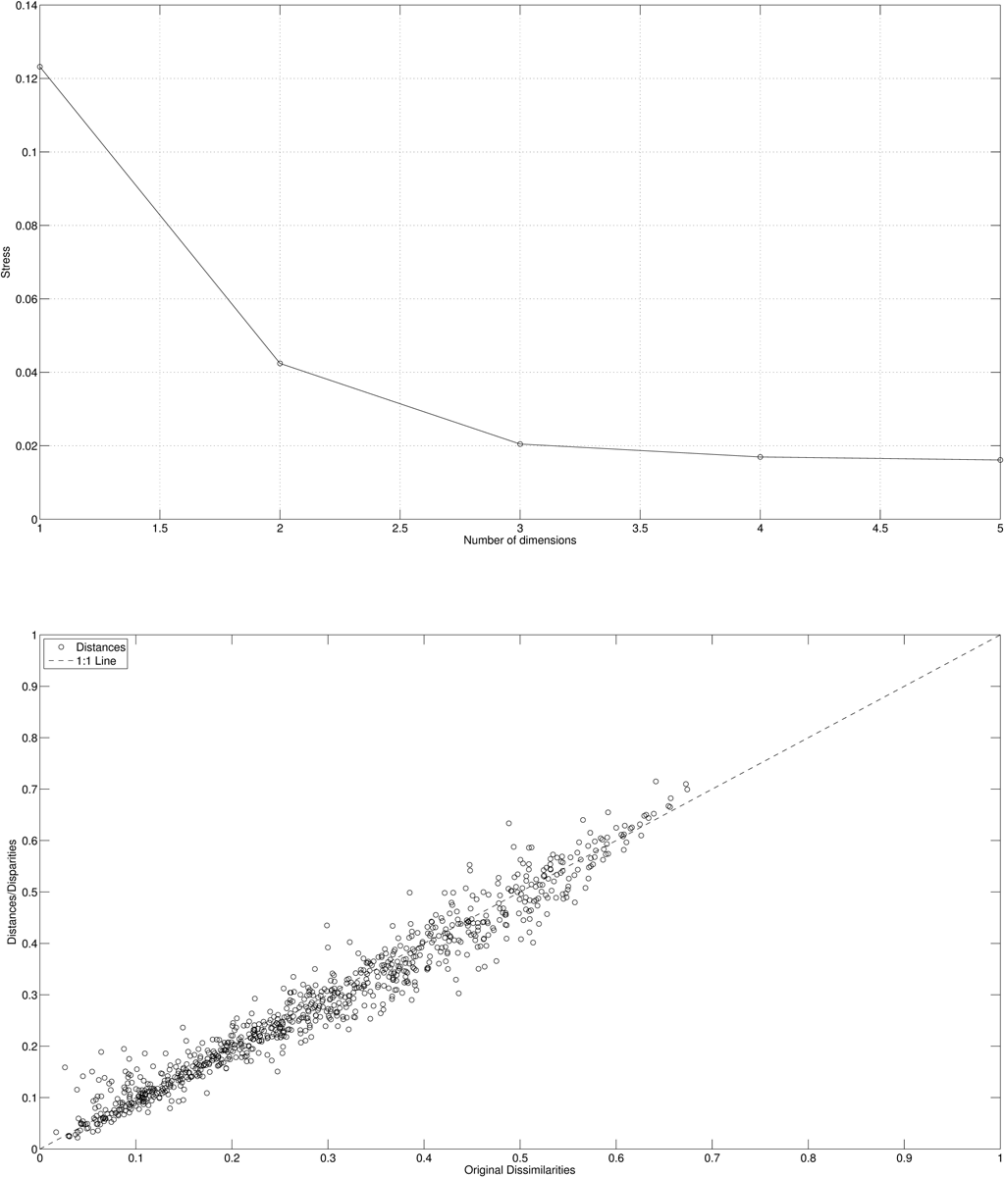
Stress plot (left) and Shepard diagram for *m* = 3 (right) using Canberra distance (4) for the *n*_*c*_ = 14 countries and 3 periods (i.e., *p* = 3), based on the 4 selected variables.

The quality of distance reproduction of the MDS plot is depicted in Fig. 8 by means of the stress and the Shepard plot. We verify that while for the Cosine-correlation *m* = 2 is sufficient for a good fit the Canberra distance requires *m* = 3.

## Conclusions

The Western world faces a great challenge from Asian partners. Current appraisals already refer that China is the world’s largest economy in 2014. From the perspective of goods and services produced China has overtaken the USA. However, the study of world economic variables for the last 36 years benefits from the application of MDS methodology, if a convergence perspective will be adopted. The analysis of a database on 14 countries over the last decades under MDS techniques proves that a large gap still separates Asian partners from converging to the North-American and Western-European developed countries, in terms of GDP per capita, economic openness, life expectancy, and tertiary education. These are are main indicators of potential warfare, economic development, and social welfare.

Looking at the presented estimations, the Western global hegemony has a closer rivalry to consider, coming from the Russian economic recovery and proximity to European partners. This paper opens new perspectives on the current global geopolitical equilibrium and peace. Recovery from banking and financial difficulties in the current downturn cycle may present a new look under this challenge revealed in the MDS results, as well as investors’ portfolio decisions after sanctions against Russia, two questions to be approached in next research.
